# AS-ULDAR: a ROS 2-based collaborative robotic platform for upper-limb rehabilitation — systems integration and pilot usability evaluation in a clinical environment

**DOI:** 10.3389/frobt.2026.1914367

**Published:** 2026-10-29

**Authors:** Chiara Alessi, Nikolas Sacchi, Cira Fundarò, Antonella Ferrara

**Affiliations:** 1 Department of Computer, Electrical, and Biomedical Engineering, University of Pavia, Pavia, Italy; 2 Neurophysiopathology Unit, Montescano Institute, Istituti Clinici Scientifici Maugeri IRCCS, Montescano, Italy

**Keywords:** graphical user interface, human-robot interaction, neurological diseases, robotic rehabilitation, usability study

## Abstract

Robotic rehabilitation can increase the intensity and personalization of upper-limb therapy, but its clinical translation is hindered by the difficulty of integrating individually validated technical components into a single clinician-ready platform. This paper presents a platform which is intended to be an Auto-calibrating System for Upper Limb Disability Assessment and Rehabilitation (AS-ULDAR), a ROS 2-based platform built around a Franka Emika Panda robot that unifies motor/cognitive assessment, sensorless force estimation, and Cartesian and Human Joint-based Trajectory Tracking within a clinician-oriented GUI. While these functionalities were individually validated in prior work, the present contribution lies in their systems-level integration and in identifying the operational barriers that emerge when deploying such a platform in a real clinical workflow, targeting adult patients with chronic neurological conditions (stroke, Parkinson’s, ALS, MCI) and mild-to-moderate upper-limb impairment. The platform was evaluated through a formative usability study: the standalone GUI by five naïve users, and the complete system by seven physiotherapists performing the full clinical workflow (no patients were involved; clinical efficacy is outside this scope). The system was technically stable throughout. Usability (SUS) was high for the GUI (87.5 ± 1.8) but markedly lower and more heterogeneous for the complete platform (54 ± 16), with feedback pointing to robot setup and workflow operation—not interface design—as the main friction points for clinical staff. These results show that integrating multiple rehabilitation functionalities is technically feasible, but that usability must be treated as a system-level property, and they provide concrete operational lessons for the clinical translation of cobot-based rehabilitation platforms.

## 1 Introduction

Neurological disorders such as stroke, Parkinson’s disease, amyotrophic lateral sclerosis (ALS), and mild cognitive impairment affect millions of people worldwide every year, often resulting in persistent motor and cognitive impairments (Dumurgier and Tzourio, 2020; Dorsey et al., 2018). Early and intensive rehabilitation is widely recognized as a key factor in promoting functional recovery and improving long-term independence, particularly when therapy is initiated soon after diagnosis or injury (Nicholson-Smith et al., 2020). However, access to rehabilitation services remains limited due to long waiting lists, shortages of specialized healthcare personnel, insufficient funding, and the limited availability of dedicated rehabilitation facilities. These challenges have motivated the development of technological solutions capable of increasing the availability, intensity, and personalization of rehabilitation treatments (Nizeyimana et al., 2022; Baroni et al., 2023).

Among these solutions, rehabilitation robotics has emerged as a promising approach to support clinicians and improve patient access to intensive therapy. Robotic systems can assist patients in performing repetitive and task-oriented movements, reduce the physical workload of physiotherapists, and provide objective measurements of patient performance throughout the rehabilitation process (Gassert and Dietz, 2018; Hao et al., 2025). Furthermore, the integration of robotic devices with interactive software platforms and exergames can enhance patient engagement and motivation, which are important factors influencing adherence to rehabilitation programs (Moulaei et al., 2023; Shang et al., 2025).

Robotic systems for upper-limb rehabilitation are commonly categorized into two main classes: exoskeleton-based devices and end-effector-based devices (Mahfouz et al., 2024). Exoskeletons are wearable robotic systems designed to align with the anatomical joints of the patient, enabling direct control of limb segments and joint trajectories. This characteristic allows accurate delivery of assistance and detailed assessment of joint-level performance (Gull et al., 2020). However, exoskeletons are often associated with high mechanical complexity, elevated manufacturing and maintenance costs, reduced scalability, and the need for careful anthropometric adjustment to accommodate different patients (Morris et al., 2023). End-effector-based systems, in contrast, interact with the patient through a single contact point, typically represented by a handle grasped by the user (Dong et al., 2022). In passive rehabilitation scenarios, the robot guides the patient’s limb along predefined trajectories, whereas in active rehabilitation the patient contributes to the movement by exerting forces on the robotic interface. Although end-effector systems provide less direct control over individual joints, they are generally simpler, more adaptable to different users, easier to deploy in clinical environments, and more cost-effective than exoskeleton-based solutions (Onfiani et al., 2024). These characteristics make them particularly attractive for large-scale rehabilitation applications and for integration with commercially available collaborative robotic manipulators.

In recent years, industrial collaborative robots (cobots) have attracted increasing attention within the rehabilitation robotics community. Compared to custom-designed rehabilitation devices, cobots benefit from industrial-grade reliability, integrated safety functionalities, widespread availability, and reduced acquisition costs resulting from large-scale production. Their flexible programming capabilities allow the implementation of different rehabilitation paradigms while simplifying maintenance and deployment procedures. Consequently, several studies have investigated the use of collaborative robots as end-effector-based rehabilitation devices for upper-limb therapy (Onfiani et al., 2024; Kyrkjebo et al., 2018).

Despite these advantages, existing cobot-based rehabilitation systems still exhibit important limitations. First, only a limited number of platforms provide integrated software environments capable of supporting clinicians through intuitive graphical user interfaces, exercise customization tools, real-time monitoring, and objective performance analysis (Casanova et al., 2025). Second, many available solutions remain tightly coupled to specific robotic hardware, making migration to different robotic platforms difficult and limiting scalability across rehabilitation centers. As a result, the deployment of robotic rehabilitation solutions often requires significant software adaptation efforts whenever a different robotic manipulator is adopted (Alessi et al., 2025a).

Another limitation concerns force measurement and interaction monitoring (Mohammadi and Dallali, 2020). Many collaborative robots used in rehabilitation applications do not include integrated force/torque sensing capabilities, and the addition of dedicated force sensors may significantly increase system cost and complexity. This aspect can hinder the adoption of robotic rehabilitation systems in resource-constrained clinical environments, despite the importance of interaction force information for patient assessment and exercise monitoring (Alessi et al., 2025b).

A further challenge relates to trajectory acquisition and reproduction. Most existing end-effector-based rehabilitation platforms allow physiotherapists to manually guide the robot and record trajectories through teach-and-repeat procedures. The recorded motion is subsequently replayed to assist patient movements (Luciani et al., 2023). While effective and easy to implement, this approach primarily captures the Cartesian trajectory of the robot end effector and does not explicitly preserve the upper-limb kinematics originally demonstrated by the therapist. Consequently, the resulting rehabilitation movement may not fully reflect the intended coordination of shoulder, elbow, and wrist joints.

Moreover, despite the growing interest in cobot-based rehabilitation, clinical evidence remains limited. A recent narrative review on the use of industrial collaborative robots for motor rehabilitation highlighted that, to date, no studies have investigated upper-limb impairments in stroke or spinal cord injury patients using industrial cobots, and that overall clinical evidence is still scarce (Raji et al., 2025). The few available pilot studies confirm this trend (Chiriatti et al., 2023): evaluated a UR5e-based rehabilitation framework involving nineteen participants, including both neurological patients and healthy controls, obtaining positive user feedback and clinically acceptable task performance. Other relevant contributions, such as (Kyrkjebo et al., 2018; Sheng et al., 2019; Lim et al., 2023; Casanova et al., 2025; Onfiani et al., 2024; Tamantini et al., 2025), assessed cobot-based systems on healthy subjects, demonstrating feasibility and potential advantages over specialist-assisted rehabilitation, but stopping short of structured clinical trials. In this context, formative usability studies involving clinicians and realistic clinical workflows represent a necessary and valuable step toward the deployment of cobot-based rehabilitation platforms in real therapeutic settings.

To address some of the limitations discussed above, our previous works introduced several complementary components for robot-assisted rehabilitation, including a ROS2-based graphical user interface for rehabilitation management (Alessi et al., 2025a), an observer-based approach for interaction-force estimation without dedicated force sensors (Alessi et al., 2025b), and a Human Joint-based Trajectory Tracking strategy enabling the generation of patient-specific rehabilitation motions from therapist demonstrations (Alessi et al., 2026). Although these solutions were individually developed and validated, they were originally presented as separate contributions and their integration into a complete rehabilitation platform suitable for clinical deployment has not been investigated.

The present work addresses this gap by presenting the Auto-calibrating System for Upper-Limb Disability Assessment and Rehabilitation (AS-ULDAR), a unified rehabilitation platform integrating all the previously developed functionalities within a single hardware and software architecture based on a Franka Emika Panda collaborative robot. The AS-ULDAR name is retained for consistency with previous publications describing the platform and its functionalities, as well as with the terminology adopted for the corresponding rehabilitation protocol. In the present work, the term “Disability Assessment” refers to the intended application of the platform and does not imply a clinical validation of its disability-assessment capabilities, which is beyond the scope of the present study. The resulting system combines motor and cognitive assessment exercises, interaction-force estimation, conventional teach-and-repeat trajectory tracking, and human-joint-based trajectory reproduction within a clinician-oriented software environment.

A comparison of representative cobot-based platforms for upper-limb rehabilitation, summarized in Table 1, further illustrates the positioning of the proposed system within the current landscape. While a few works have addressed individual aspects of robot-assisted rehabilitation, such as gamified exercise frameworks and compatibility with a wide class of cobots via ROS framework (Casanova et al., 2025) or sensorless force estimation (Brahmi, 2026), none of the reviewed systems simultaneously integrates a clinician-friendly GUI, sensorless interaction force estimation, cognitive exercises, and validation in a real clinical environment. Notably (Khan et al., 2023), proposed a telerehabilitation framework compatible with multiple industrial end-effector robots and integrating an AR-based GUI supporting both motor and cognitive exercises. However, the system was specifically designed for remote therapy rather than in-presence rehabilitation, and has not been validated in a structured clinical environment, limiting its direct comparability with the systems reviewed above. Additionally, to the best of our knowledge, no existing platform implements a joint-based trajectory tracking task that accounts for inter-subject anthropometric differences during exercise reproduction, a feature that distinguishes the proposed system from conventional Cartesian-based approaches.

**TABLE 1:** Comparison of representative cobot-based platforms for upper-limb rehabilitation.

System	Robot	Clinical GUI	Sensorless force Est	Joint-based TT	Cognitive exercises	Clinical-setting testing	Software framework
(Casanova et al., 2025)	UR family	Yes	No	No	Yes	Healthy subjects only	ROS
(Fernandes et al., 2020)	UR3	No	Not specified	No	No	Not specified	Not specified
(Chiriatti et al., 2023)	UR5e	No	No	No	No	Yes (neurological patients)	Not specified
(Lim et al., 2023)	Collaborative robot	Yes	No	No	Yes	Healthy subjects only	ROS
AS-ULDAR (proposed)	Franka Emika Panda	Yes	Yes	Yes	Yes	Yes (therapists only, no patients)	ROS 2

Thus, the main contribution of this work is the integration of previously developed components into a complete rehabilitation platform and the evaluation of its usability in realistic operating conditions. To this end, the platform was deployed in a clinical environment and assessed through a formative usability study involving physiotherapists performing the complete rehabilitation workflow. In parallel, the graphical user interface was evaluated independently by users without robotics expertise in order to assess its usability separately from the robotic hardware.

The objectives of this study are twofold: (i) to present the complete AS-ULDAR platform and its integrated functionalities within a structured clinical workflow, and (ii) to investigate the usability, operational feasibility, and technical reliability of the proposed system in clinical rehabilitation settings. Specifically, to address the second objective, this paper answers three main Research Questions:

RQ1 (Technical Reliability and Operational Execution): Is the integrated platform technically reliable when operated by physiotherapists in a clinical environment, as evidenced by the absence of system crashes, software execution failures, or adverse technical events during the usability protocol?

RQ2 (System vs. Standalone GUI Usability): How does the perceived usability of the standalone control interface compare to that of the fully integrated robotic platform when operated by clinical staff in a hospital setting?

RQ3 (Lessons Learned and Design Guidelines): What are the primary usability barriers, operational insights, and design guidelines that emerge from physiotherapists’ qualitative feedback to inform future clinical robotic deployments?

It is important to emphasize that while the AS-ULDAR platform is designed for patient treatment and an ongoing clinical trial is currently collecting data on neurological subjects, evaluating clinical efficacy and patient-level safety is beyond the scope of this paper. The primary contribution of this work lies in presenting the overall platform architecture, verifying its technical integration and operational reliability during therapist interaction, mapping the clinical workflow usability gap, and extracting design guidelines from end-user physiotherapists.

The remainder of this paper is organized as follows. Section 2 describes the materials and methods. In particular, Section 2.1 presents the AS-ULDAR platform, describing its hardware and software architecture as well as the interactions among its components. Section 2.2 details the main rehabilitation tasks integrated into the platform (CO-PTP, CTT, and HJTT). Section 2.3 presents the underlying low-level robot control strategies used during the execution of the rehabilitation tasks, while Section 2.4 outlines the patient-robot interaction paradigms. Section 2.5 describes the target user population and the clinical rehabilitation workflow. Section 2.6 details the experimental procedures and evaluation protocols designed to address the research questions (**RQ1**–**RQ3**). Section 3 presents the empirical findings from both technical reliability logs and usability assessments. Finally, Section 4 discusses these results, contextualizing the findings, outlining clinical implications, and addressing study limitations alongside future research directions.

## 2 Materials and methods

### 2.1 The AS-ULDAR platform

The overall system is composed of interconnected hardware and software components with which the involved users, namely, physiotherapists and patients, interact during rehabilitation sessions, as depicted in Figure 1. In particular, the physical setup includes a Franka Emika Panda collaborative robotic manipulator equipped with its dedicated controller, a safety anti-tip table ensuring stable installation of the robot, a custom-designed end-effector (EE) handle to be grasped by the patient, and a computer workstation with a display unit. In addition, multimodal motion acquisition is enabled through XSens MTw Awinda wearable inertial sensors.

**FIGURE 1:**
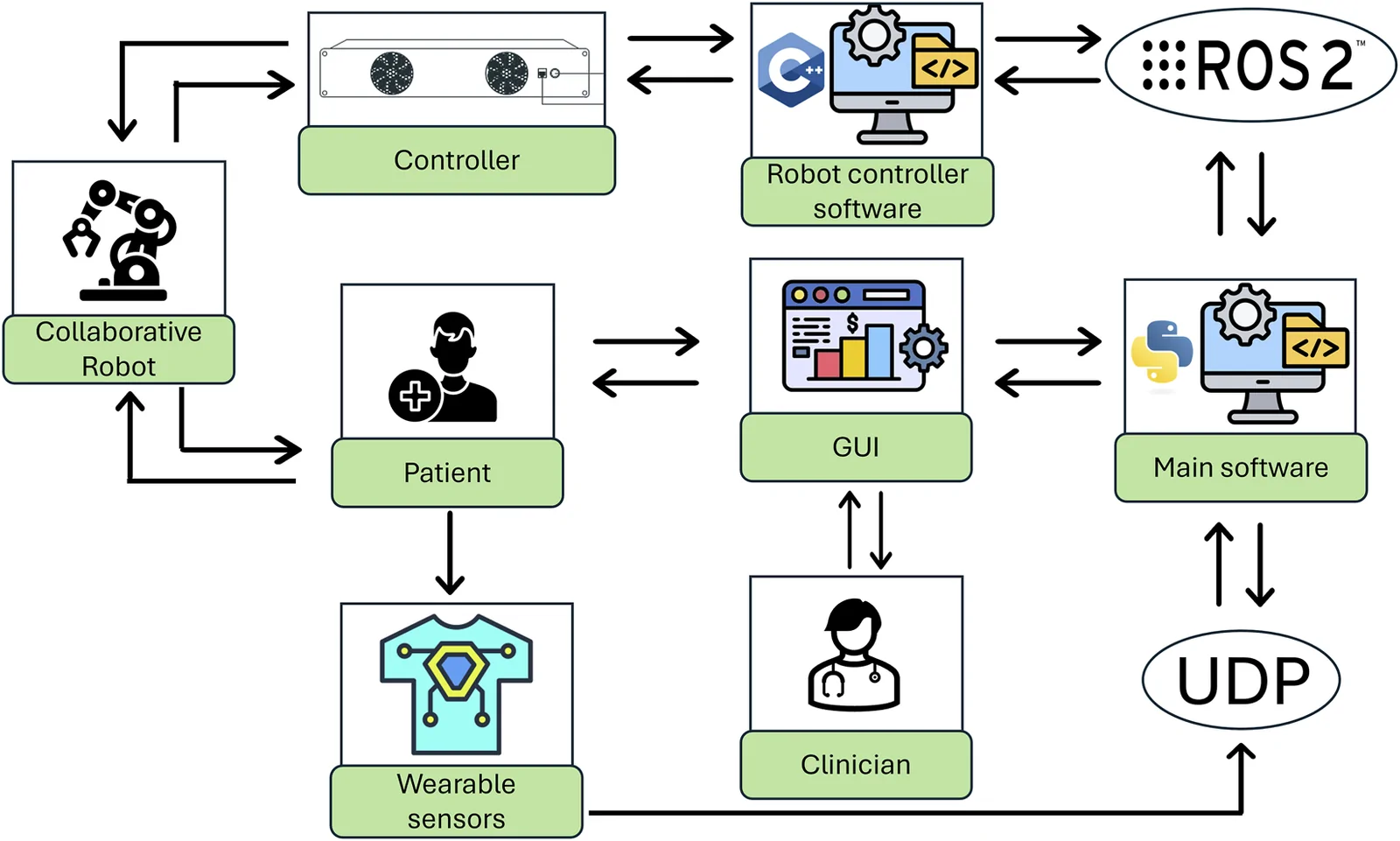
System operation diagram including hardware and software components, communication frameworks, and users.

The workstation hosts the software modules responsible for robot control, data acquisition and processing, and rehabilitation exercise execution. These functionalities are integrated within a graphical user interface (GUI) specifically designed to support clinical operation, exercise configuration, and session management. Communication among the various hardware and software components is implemented through the Robot Operating System 2 (ROS 2) middleware, which provides a modular and distributed communication framework based on publishers, subscribers, services, and actions. This architecture enables seamless information exchange between the robot controller, wearable sensors, GUI, and the different software modules, while ensuring scalability, interoperability, and real-time data sharing across the entire rehabilitation platform.

In the following subsections, the individual components of the architecture are described in detail.

#### 2.1.1 Platform hardware

##### 2.1.1.1 The Franka Emika Panda robot

At the core of the AS-ULDAR platform lies the Franka Emika Panda collaborative robot, a lightweight 7-degree-of-freedom (DoF) serial manipulator specifically designed for applications involving direct physical human–robot interaction. Owing to its kinematic redundancy, integrated joint torque sensing, low-friction actuation, and high backdrivability, the Panda is particularly well suited for upper-limb rehabilitation tasks, where safe and compliant interaction with the patient is of paramount importance. A view of the robotic platform is reported in Figure 2.

**FIGURE 2:**
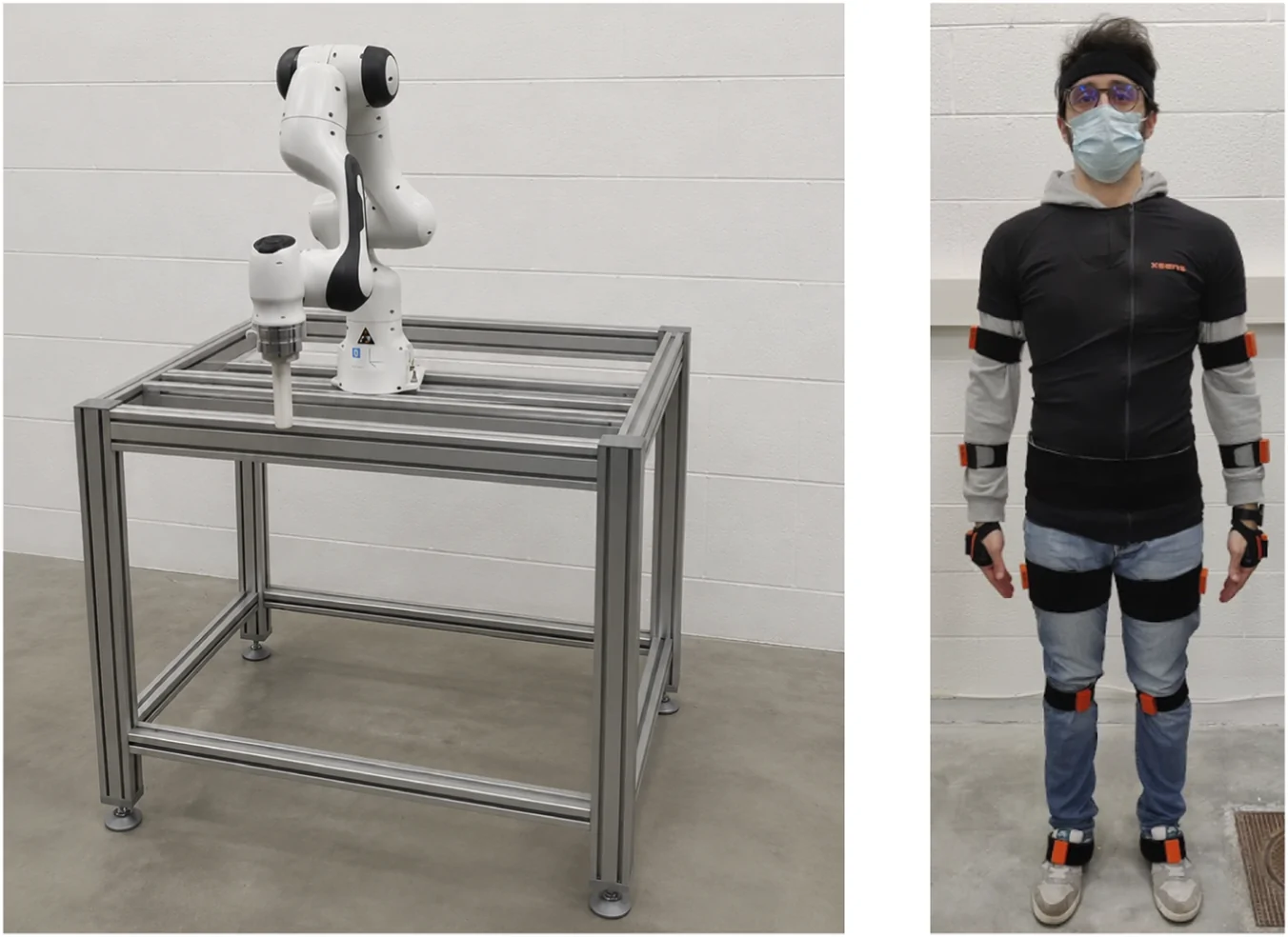
Main hardware components of the AS-ULDAR platform: the Franka Emika Panda robotic manipulator mounted on the custom support structure (left) and the XSens MTw Awinda wearable inertial sensing system (right).

The manipulator provides a workspace radius of approximately 855 mm, a payload capacity of 3 kg, and a Cartesian repeatability of 0.1 mm. Furthermore, torque sensors embedded in each joint enable accurate estimation of external interaction forces and facilitate the implementation of advanced torque-based control strategies. The robot is interfaced through the Franka Control Interface (FCI), which provides access to real-time measurements and low-level control functionalities at a frequency of 1 kHz. Thanks to its redundant kinematic structure, the robot can achieve highly dexterous motions while avoiding kinematic singularities and joint limits during rehabilitation tasks. Furthermore, the platform can be seamlessly integrated within ROS 2-based software architectures, facilitating interoperability with external sensors, graphical interfaces, and control modules.

To guarantee safe and stable operation, the robot is mounted on a dedicated support structure consisting of a frame realized using anodized aluminum profiles, providing high structural rigidity while preserving flexibility in system reconfiguration. Adjustable leveling feet enable accurate alignment of the platform with respect to the floor, ensuring repeatable calibration and reducing the effects of vibrations during rehabilitation exercises.

Patient interaction with the robotic manipulator is achieved through a custom-designed end-effector handle. The device was developed using the computer-aided design (CAD) tool Tinkercad, and manufactured through fused deposition modeling (FDM) 3D printing using polylactic acid (PLA). The handle geometry was iteratively refined to improve ergonomics, comfort, and grasp stability during prolonged rehabilitation sessions. When patients are unable to maintain a stable grasp due to severe motor impairments or spasticity, dedicated assistive devices, such as spasticity gloves, can be employed to ensure a secure mechanical coupling between the patient’s hand and the robot end-effector.

##### 2.1.1.2 XSens MTw Awinda wearable sensors

To complement the information provided by the robotic manipulator, the AS-ULDAR platform integrates a wearable motion capture system based on XSens MTw Awinda inertial sensors, depicted in the right part of Figure 2. The system enables non-invasive monitoring of patient movements and provides additional kinematic measurements that can be exploited for motion analysis, patient assessment, and estimation algorithms.

The XSens MTw Awinda is a wireless inertial measurement unit (IMU) integrating a tri-axial accelerometer, tri-axial gyroscope, and tri-axial magnetometer within a compact wearable device. Through proprietary sensor-fusion algorithms, each sensor provides real-time estimates of its orientation with respect to a global reference frame, together with raw inertial measurements. The wireless communication architecture allows multiple sensors to be synchronized and acquired simultaneously without restricting the patient’s movements during rehabilitation exercises. In the current implementation, the sensors are attached to selected segments of the patient’s upper limb using adjustable straps, as shown in Figure 2.

The MTw Awinda sensors communicate with a dedicated Awinda Station connected to the workstation hosting the AS-ULDAR software. Through a custom ROS two interface, the measurements are acquired in real time and made available to the different software modules of the platform. This integration enables seamless synchronization between wearable sensing, robot state measurements, and rehabilitation task execution. In the proposed rehabilitation paradigm, such sensors can be worn by both the therapist when recording a trajectory in the human arm joint angle space, and by the patient during the assessment phase.

#### 2.1.2 Software architecture and graphical user interface (GUI)

The AS-ULDAR platform includes a dedicated graphical user interface (GUI) designed to support the clinical use of the rehabilitation system. The GUI represents the main interaction layer between the physiotherapist and the robotic platform, allowing the operator to configure rehabilitation exercises, manage patient data, start and stop robot-assisted tasks, monitor the execution of each session, and inspect the obtained results. From the software point of view, the GUI is implemented as a ROS two node, which communicates with the robot and the remaining software modules through predefined ROS two topics.

The GUI has been developed to hide the complexity of the underlying robotic architecture from the clinical user. In particular, the physiotherapist is not required to directly interact with low-level robot controllers or command-line tools. Instead, the main robot operating tasks can be selected through buttons and dedicated panels. These commands are then translated into ROS two messages by the GUI node, which publishes them on the corresponding control topics. In this way, the clinician can launch different rehabilitation exercises through a unified interface.

The interface, schematically represented in Figure 3 and partially shown in Figure 4, is organized into three main functional areas:

- *Patient Management Area*: which allows the operator to create new patient profiles, select existing patients, and associate each rehabilitation session with the corresponding clinical record. This functionality is required to ensure that exercise parameters and performance indicators are stored consistently and can be retrieved across different sessions.
- *Trajectory Recording Area*: used when the clinician needs to define a new reference motion through a teaching-by-demonstration procedure. During this phase, the robot is set in hand-guiding mode and the clinician manually moves the EE along the desired path. The acquired motion together with the corresponding task-space information when required, is saved in the local storage and can later be used during trajectory-tracking rehabilitation exercises.
- *Individual Monitoring and Therapy Area*: supports the execution and evaluation of patient-specific rehabilitation exercises. This area includes an exercise preparation panel, an execution panel, a results panel, and a details panel. In the preparation panel, the clinician selects the type of exercise, configures the relevant parameters, and loads previously recorded trajectories or patient-specific data. The execution panel is used to start, supervise, and stop the ongoing exercise. The results panel summarizes the main performance indicators obtained during the previous sessions, while the details panel provides additional information related to the patient.

**FIGURE 3:**
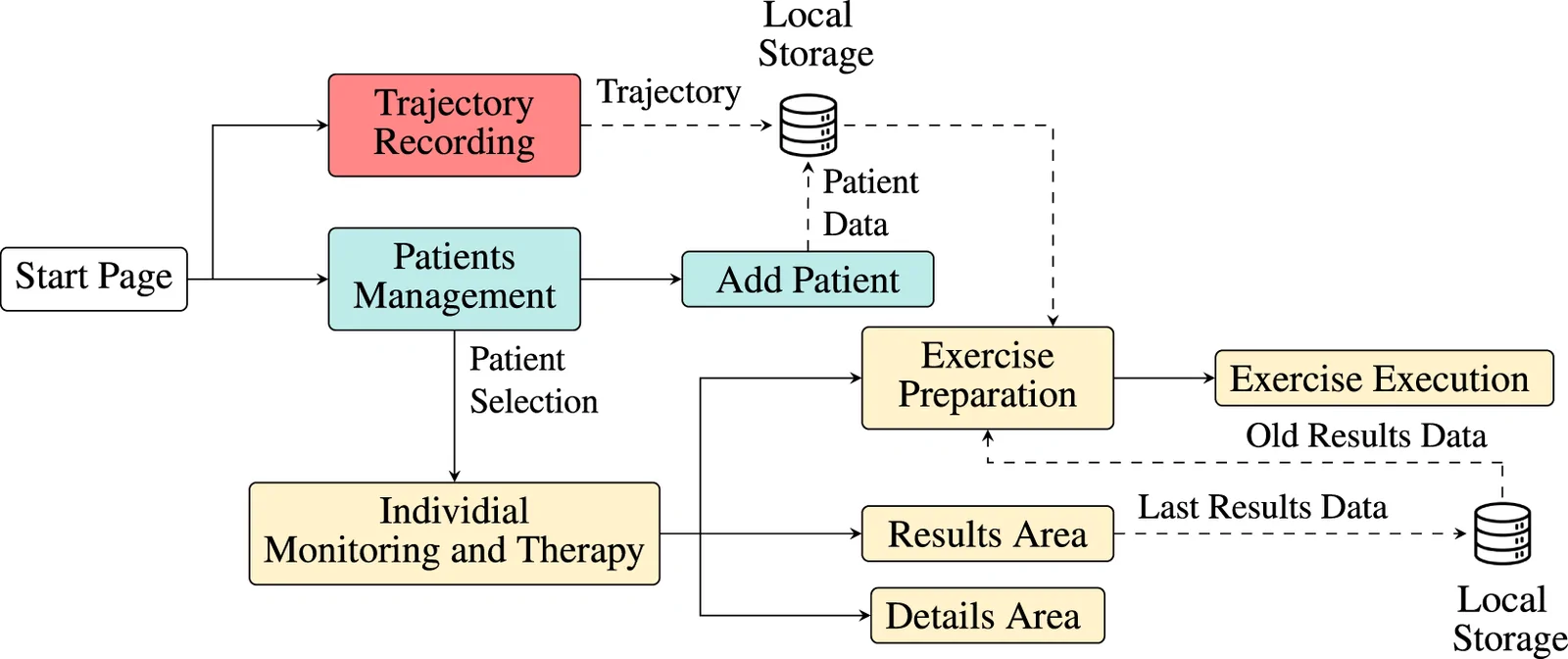
Diagram of the GUI structure. The dashed lines represent interactions with the local storage.

**FIGURE 4:**
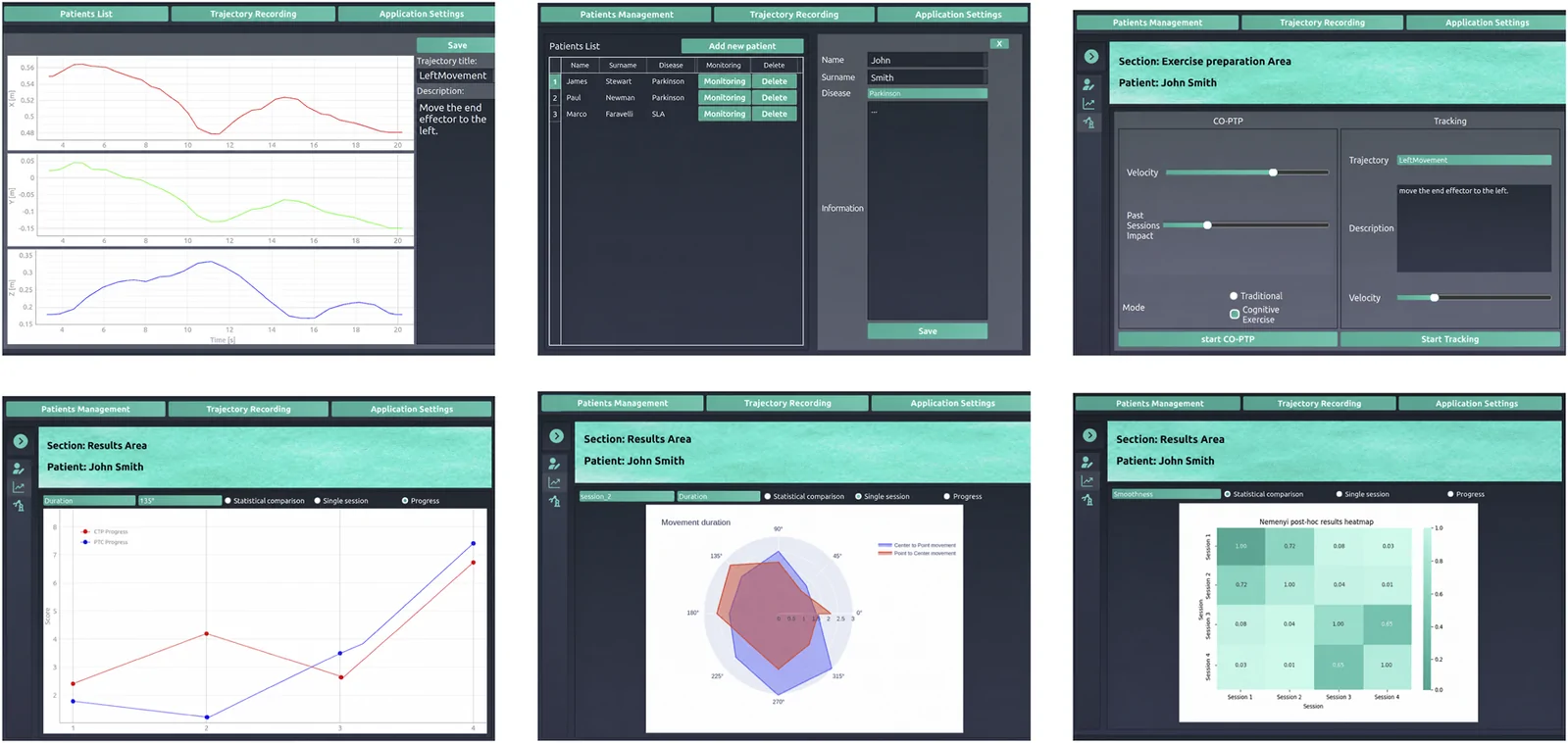
Screenshots of some parts of the GUI.

During operation, the GUI continuously receives robot-state information, including joint positions, joint velocities, EE pose, and end-effector velocity. These data are used both for real-time visualization and for the computation of quantitative metrics describing the patient’s motor performance. At the same time, the GUI sends control commands and, when required, torque references to the robot control node. Therefore, the GUI is not merely a visualization tool, but an active component of the rehabilitation architecture, coordinating user interaction, exercise execution, data storage, and robot control.

All patient data, recorded trajectories, exercise parameters, and session results are stored locally on the workstation. This design choice simplifies deployment in clinical environments and allows the clinician to retrieve previous results when configuring new rehabilitation sessions. In particular, past performance can be used to adapt exercise difficulty, select target directions, or tune cognitive-exercise parameters, thus enabling a patient-specific rehabilitation workflow.

### 2.2 Functionalities of AS-ULDAR

The platform provides three main rehabilitation functionalities (tasks): Center-Out Point-to-Point (CO-PTP), Cartesian Trajectory Tracking (CTT), and Human Joint Trajectory Tracking (HJTT). To ensure a clear and unambiguous distinction between robotic execution and clinical interaction, each task is explicitly characterized in terms of:

- Robot Control Strategy: Defines the underlying low-level closed-loop controller governing the robotic manipulator dynamics (e.g., Zero-Impedance Control via dynamic torque compensation, or Suboptimal Second-Order Sliding Mode trajectory tracking).
- Patient-Robot Interaction/Assistance Paradigm: Defines the physical coupling and mutual roles of the patient and the robot during the task execution. (e.g., Unassisted/Active motion vs. Fully Assisted/Robot-Guided motion).

The association between tasks, Robot Control Strategies, and Patient-Robot Interaction/Assistance Paradigm is defined in Table 2, while a more precise description of the control strategies and assistance paradigms is provided in Sections 2.3 and 2.4, respectively.

**TABLE 2:** Platform tasks, underlying control strategies, and interaction paradigms.

Rehabilitation task	Task description	Robot control Strategy	Patient-robot interaction Paradigm
CO-PTP *(TM and CEM)*	Active reaching toward 8 coplanar target points. Neither spatial path nor speed profile is prescribed	Zero-Impedance Control	Robot: Passive (transparent) Patient: Active *Paradigm:* Unassisted (no assistance/resistance)
CTT *(Recording)*	Demonstration and manual recording of 3D Cartesian end-effector trajectories	Zero-Impedance Control	Robot: Passive (transparent) Therapist: Active *Paradigm:* Manual guidance (no assistance/resistance)
CTT *(Reproduction)*	Reproduction of Cartesian end-effector motion profiles with prescribed spatial path $\left(R^{3}\right)$ and timing	SSOSM Control	Robot: Active Patient: Passive *Paradigm:* Fully Assisted/Robot-Guided
HJTT *(Reproduction)*	Reproduction of human joint kinematic trajectories with prescribed joint path $\left(Q_{H}\right)$ and timing	SSOSM Control	Robot: Active Patient: Passive *Paradigm:* Fully Assisted/Robot-Guided

Detailed formulation of closed-loop control algorithms is reported in Section 2.3.

#### 2.2.1 Center out point to point

One of the most common exercises employed in upper-limb rehabilitation is the so-called *Center-Out Point-to-Point* (CO-PTP) task. During this exercise, the patient is asked to grasp the robot EE through the dedicated handle and perform a Reaching Movement (RM), i.e., move it from a central starting position toward one of several targets arranged along a circumference centered at the starting point. Once the selected target is reached, the patient is required to return to the initial position. In the AS-ULDAR system, the CO-PTP task comprises eight coplanar target points arranged along a circle, as illustrated in Figure 5. They are defined within a horizontal plane positioned at chest height in front of the patient. The robotic manipulator does not impose any physical or virtual hardware constraints on the end-effector motion; thus, the patient remains mechanically free to execute a three-dimensional reaching trajectory. However, during clinical execution, the task is designed to be performed over a physical horizontal supporting surface (e.g., a table).

**FIGURE 5:**
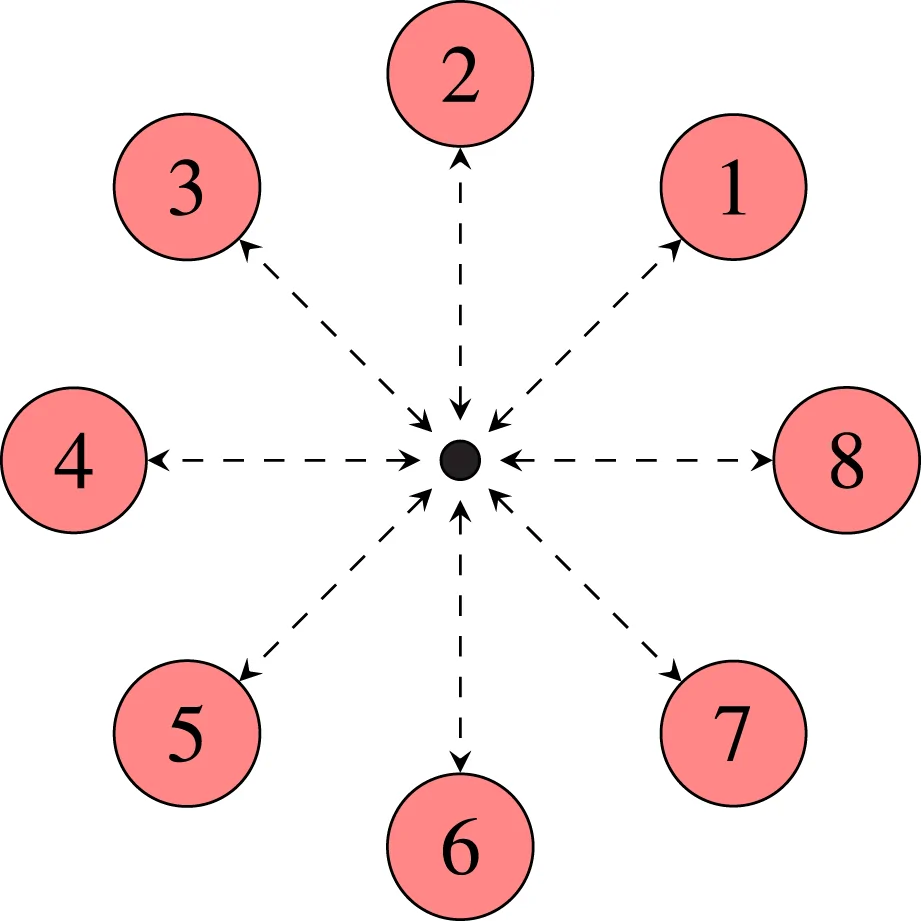
The eight targets of the CO-PTP exercise with the associated ideal movement directions. The black circle in the center denotes the start position.

Besides its rehabilitation role, the CO-PTP task is widely adopted as an assessment tool for evaluating upper-limb motor performance. In particular, by requiring movements along multiple directions, it enables the identification of direction-dependent motor impairments, which are commonly observed in post-stroke patients due to muscular weakness, abnormal synergies, or reduced motor coordination. To this end, the AS-ULDAR platform computes a set of Quality Metrics (QMs) for each target and the corresponding reaching movement. These metrics provide quantitative information about the patient’s motor capabilities in different regions of the workspace and can be employed to monitor rehabilitation progress over time, identify specific deficits, and support the customization of rehabilitation protocols.

Let $p_{c}\in R^{2}$ denote the center position, $p_{i}^{⋆}\in R^{2}$ the position of the i -th target, and p(t) the end-effector position during the corresponding Reaching Movement (RM), which consists of a center-to-target movement followed by a target-to-center movement. Moreover, let $T_{i}$ denote the duration of the RM, $v\left(t\right)=\|\dot{p}\left(t\right)\|$ the end-effector speed, and $t_{i}^{⋆}$ the instant at which the end-effector first reaches the i -th target. Finally, let |F(t)| be the amplitude of the force exerted on the EE by the patient. The QMs considered in the AS-ULDAR system are summarized in Table 3. The kinematic and dynamic features computed by AS-ULDAR (e.g., movement duration, smoothness, reaching error) have been shown in the literature to correlate with standard clinical scales for upper-limb disability, such as the Fugl Meyer scale. This supports the use of the CO-PTP task as a quantitative, technology-assisted disability assessment tool, complementary to standard clinical scales (Zollo et al., 2011). This existing evidence motivates the long-term ambition of the platform, reflected in its name, to support quantitative, technology-assisted disability assessment as a complement to standard clinical scales. However, this assessment capability has not yet been validated within AS-ULDAR itself, since the present study did not involve neurological patients or comparisons against clinical disability scores. Establishing this correlation directly on the platform is one of the objectives of the ongoing clinical trial (protocol number [57875/2025]), and constitutes a necessary step before any disability-assessment claim can be substantiated for the proposed system.

**TABLE 3:** QMs associated with the i -th target.

**QM**	Description	Formula
$d_{i}$	Duration of the RM	$T_{i}$
$\epsilon_{i}$	Target and center reaching error	$\left\|p_{i}^{⋆}-p\left(t_{i}^{⋆}\right)\right\|+\left\|p_{c}-p\left(T_{i}\right)\right\|$
$v_{i}$	Mean EE speed during the RM	$T_{i}^{-1}\int_{0}^{T_{i}}v\left(t\right)\;dt$
$s_{i}$	Smoothness	${\left(\max_{t\in \left[0,T_{i}\right]}v\left(t\right)\right)}^{-1}v_{i}$
$f_{i}$	Peak force	$\max_{t\in \left[0,T_{i}\right]}|F\left(t\right)|$

##### 2.2.1.1 Exercise modalities

The CO-PTP exercise can be executed in two different modalities, namely, the *Traditional Mode* (TM) and the *Cognitive Exercise Mode* (CEM) (Alessi et al., 2025a). While TM is primarily intended for motor rehabilitation and assessment, CEM additionally introduces a memory component, thereby providing a combined motor-cognitive training activity.

In TM, the patient is instructed to perform a RM toward a specified target i∈{1,…,8} . As previously described, a RM consists of a center-to-target movement followed by a target-to-center movement. The system continuously monitors the end-effector position and velocity and automatically detects the completion of the RM. Specifically, a RM is considered completed when the end-effector speed falls below $5\times 10^{-4}\;m/s$ and its distance from the center position is lower than 0.03 m . Upon completion, the patient is instructed to perform the next RM. The exercise terminates once all targets have been visited.

Conversely, in CEM, the patient is required to memorize and subsequently execute a sequence of target-directed RMs. Before the beginning of the exercise, the target sequence is displayed through the GUI. The sequence length and display speed are configurable by the clinician. Unlike TM, the target sequence is generated according to the patient’s performance during previous rehabilitation sessions. This choice is motivated by the fact that neurological disorders such as stroke frequently produce direction-dependent motor impairments. Consequently, directions associated with poorer motor performance should be exercised more frequently than those associated with better performance. For this reason, the selection of the targets in the computation of the sequence depends on the performance of the patient in different directions during previous rehabilitation sessions. This performance-driven adaptation of exercise difficulty constitutes the auto-calibrating mechanism referenced in the platform’s name. More information regarding the computation of the sequence can be found in (Alessi et al., 2025a).

##### 2.2.1.2 Interaction force estimation

During CO-PTP execution, interaction forces are estimated using a decentralized bank of Integral Sliding Mode (ISM) Unknown Input Observers, with one observer per robot joint (Utkin and Shi, 1996), thus without requiring any dedicated force/torque sensor. The estimated interaction force is used for real-time monitoring of patient effort and for the computation of force-based performance indicators during rehabilitation exercises. The formulation and experimental validation of the ISM observers were previously presented in (Alessi et al., 2025b), where their estimation accuracy was characterized in terms of RMSE, including the observed asymmetry across estimation axes and its interpretation with respect to clinically and practically acceptable error levels. In that work, the suitability of the estimation accuracy was discussed in relation to the intended use of the observer, namely, patient-effort estimation rather than high-precision haptic rendering. In the present work, the observer is therefore not re-validated as an independent methodological contribution, but considered as an integrated component of the overall rehabilitation platform.

#### 2.2.2 Trajectory tracking

Among the various rehabilitation tasks available for upper-limb therapy, *Trajectory Tracking* (TT) exercises are widely employed (e.g., (Furuhashi et al., 2009; Lum et al., 2004; Nielsen et al., 2017)), particularly during passive rehabilitation phases. In these exercises, the robotic system guides the patient’s limb along predefined motions designed by the therapist, thereby promoting motor recovery while preventing potentially harmful movements and excessive mechanical loading. From a clinical standpoint, passive therapy is predominantly indicated in the acute and early subacute stages of neurological recovery, when patients exhibit severe motor deficits and cannot voluntarily initiate movement. In this context, fully assisted guidance acts as a foundational intervention to preserve joint mobility, mitigate soft-tissue contractures, and deliver essential proprioceptive input, thereby establishing a physiological baseline to support the subsequent transition toward active or adaptive rehabilitation regimes (Wang et al., 2025; Zhang et al., 2018).

##### 2.2.2.1 Cartesian Trajectory Tracking

A common approach for defining TT exercises relies on the direct demonstration of the desired motion by the therapist, as schematically illustrated in Figure 6. During the exercise-design phase, the therapist physically moves the robot EE along the desired path while the corresponding Cartesian pose trajectory $x_{e,r,⋆}^{b}:\;R_{\geq 0}\to R^{6}$ is recorded. Subsequently, during the rehabilitation session, the recorded Cartesian trajectory is reproduced by the robotic system. More specifically, the desired Cartesian trajectory $x_{e,r,⋆}^{b}\left(t\right)$ is converted into a desired joint-space trajectory $q_{r}^{⋆}:\;R_{\geq 0}\to R^{7}$ through the solution of an inverse kinematics (IK) problem, solved via numerical algorithm such as the Newton-Raphson method (Goldenberg et al., 2003). The resulting joint references are then tracked by the robot low-level controllers, causing the patient’s hand, rigidly attached to the EE, to follow the same Cartesian motion demonstrated by the therapist. Consequently, the hand trajectory satisfies $x_{h}^{b}\left(t\right)=x_{e,r,⋆}^{b}\left(t\right)$ , where $x_{h}^{b}$ represents the pose of the hand of the patient. Owing to the fact that the exercise is defined and reproduced in the Cartesian space, this rehabilitation task is commonly referred to as *Cartesian Trajectory Tracking* (CTT).

**FIGURE 6:**
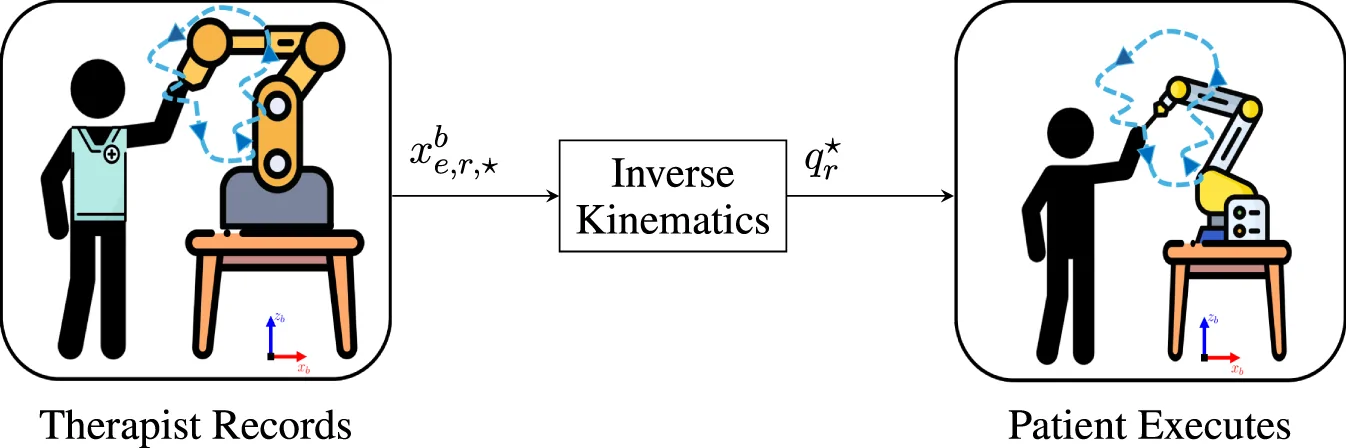
Standard workflow for CTT exercise demonstration and execution. $x_{e,r,⋆}^{b}$ denotes the pose of the robot EE recorded during the demonstration phase, while $q_{r}^{⋆}$ denotes the corresponding robot joint configuration that achieves this pose and is obtained through inverse kinematics.

Although straightforward and clinically intuitive, CTT implicitly assumes that the therapist and the patient share similar upper-limb kinematic characteristics. In practice, differences in limb lengths, joint mobility, and anthropometric proportions may cause the same Cartesian trajectory to correspond to substantially different joint configurations. As a result, the patient may experience joint motions, muscle activation patterns, and mechanical loads that differ significantly from those originally intended by the therapist, potentially reducing the effectiveness and repeatability of the rehabilitation treatment.

##### 2.2.2.2 Human joint-based trajectory tracking

To overcome the limitations of conventional CTT exercises, the AS-ULDAR also allows to perform *Human Joint-based Trajectory Tracking* (HJTT) exercises (Alessi et al., 2026). Unlike CTT, where the rehabilitation objective is specified in terms of the hand trajectory, HJTT defines the rehabilitation task directly in the patient’s joint space. Specifically, the objective is to guide the patient such that the upper-limb joint coordinates $q_{h}$ follow a prescribed trajectory $q_{h}^{⋆}:\;R_{\geq 0}\to Q_{p}^{h}$ . The recording phase of HJTT differs significantly from that of CTT. Rather than recording the motion of the robot EE, the therapist performs the desired rehabilitation exercise while the corresponding human joint trajectory is measured. Therefore, the quantity stored in the exercise database is the trajectory $q_{h}^{⋆}\left(t\right)$ rather than a Cartesian end-effector trajectory. Since the objective is to record a human joint trajectory rather than a robot motion, the robotic manipulator is not required during the recording and the joint coordinates are acquired using the XSens wearable inertial sensors in the right part of Figure 2. The overall workflow is schematically illustrated in Figure 7.

**FIGURE 7:**
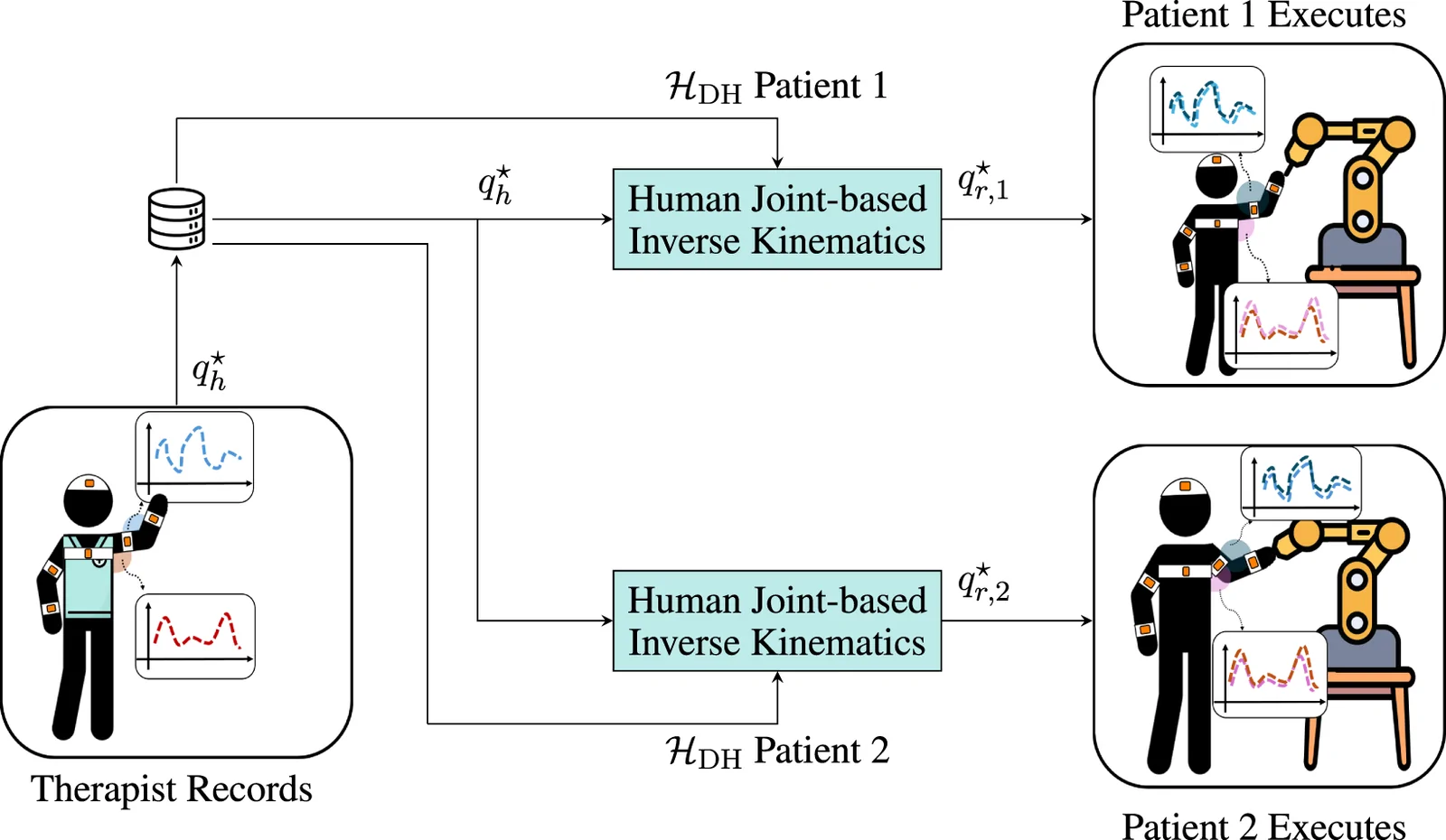
Block diagram illustrating the trajectory recording performed by the therapist and the reference generation for two patients with different limb morphologies.

The key advantage of this approach is that the same therapeutic motion can be transferred to patients characterized by different anthropometric parameters. In fact, the recorded trajectory $q_{h}^{⋆}$ is independent of the specific robot configuration and can be converted into patient-specific robot trajectories through a Human Joint-based Inverse Kinematics (HJIK) algorithm. The HJIK formulation is based on the kinematic models of both the robotic manipulator and the human upper limb, and on the differential kinematic relationship linking variations in the robot joint space to variations in the human joint space. This relationship is derived under the assumption that the relative pose between the patient’s hand and the robot end-effector remains constant during the exercise. This assumption is designed to hold both for healthy participants firmly grasping the handle and for patients using dedicated assistive grasping devices (e.g., spasticity gloves), which are specifically intended to secure a rigid mechanical coupling between the patient’s hand and the end-effector. Cases in which this coupling cannot be considered strictly rigid (for instance due to residual slippage within the assistive device) are not currently addressed by the HJIK formulation and represent a limitation to be investigated in future work. Based on this relationship, the recorded human joint trajectory can be converted into a robot trajectory adapted to the anthropometric characteristics of the patient. Regarding convergence and computational complexity, HJIK follows a damped least-squares (Levenberg–Marquardt-type) Jacobian formulation (Wampler, 1986), where local convergence near the solution is guaranteed under standard Jacobian-conditioning assumptions, while global convergence is not guaranteed in general, as with any method in this class. Each frame is warm-started from the previous frame’s solution, exploiting the temporal continuity of the motion. Letting n denote the number of degrees of freedom of each kinematic chain (4 for the human arm, seven for the robot), the per-iteration cost is O(n) plus a fixed-size matrix inversion; the overall trajectory cost is therefore O(N.K), where N is the number of frames and K is the number of iterations required per frame to reach convergence. Across five recorded trials (2624 frames, 8–10 s each), the algorithm converged in a median K = 2 iterations per frame (mean 1.9, max 3) with a 100% success rate, processing each frame in 0.7 m on a single generic CPU core.

More details regarding the design of the HJIK algorithm and its experimental validation can be found in (Alessi et al., 2026). In particular, detailed joint-by-joint RMSE values, the statistical significance (p-values) of the comparison against the traditional method CTT are reported. Additionally, a video illustrating the experimental protocol adopted to validate the proposed method is available^1^.

### 2.3 Control strategies and system dynamics

To ensure operational transparency, modularity, and physical safety during human–robot interaction, the AS-ULDAR control framework is implemented directly at the joint torque level, within the ROS 2 Control framework, executing real-time state feedback and command update loops at a sampling frequency of 1 kHz.

Consider the dynamic model of an n -degree-of-freedom ( n=7 for the Franka Emika Panda) serial manipulator interacting with the user:

$$M\left(q\right)\ddot{q}+C\left(q,\dot{q}\right)\dot{q}+g\left(q\right)=\tau_{\text{cmd}}+\tau_{\text{ext}},$$ (1)

where $q,\dot{q},\ddot{q}\in R^{n}$ are the joint position, velocity, and acceleration vectors, respectively; $M\left(q\right)\in R^{n\times n}$ is the symmetric positive-definite inertia matrix; $C\left(q,\dot{q}\right)\dot{q}\in R^{n}$ collects the Coriolis and centrifugal terms; $g\left(q\right)\in R^{n}$ is the gravity torque vector; $\tau_{\text{cmd}}\in R^{n}$ is the commanded joint torque vector; and $\tau_{\text{ext}}\in R^{n}$ collects the external joint torques induced by the interaction force applied by the therapist or the patient at the end-effector.

Depending on the functional task selected through the GUI, the control architecture switches between two torque-level strategies.

#### 2.3.1 Zero-impedance control

This control strategy is used whenever the robot must offer minimal resistance to externally imposed motion: during Trajectory Recording (hand-guided demonstration by the therapist) and during CO-PTP exercises (active reaching movements performed by the patient). The controller compensates the non-inertial dynamic terms of (1) without introducing any position- or velocity-tracking feedback:

$$\tau_{\text{cmd}}=C\left(q,\dot{q}\right)\dot{q}+g\left(q\right).$$ (2)

Substituting (Equation 2) into (Equation 1) yields the closed-loop dynamics

$$M\left(q\right)\ddot{q}=\tau_{\text{ext}},$$ (3)

i.e., the robot behaves as a free inertial system driven solely by the interaction torque. In the absence of external interaction $\left(\tau_{\text{ext}}=0\right)$ , the manipulator remains at rest with zero acceleration; when perturbed, it offers no active resistance beyond its own inertia and exhibits no restoring behavior toward a reference configuration, resulting in a fully backdrivable structure. This regime is also what enables the interaction-force estimation described in Section 2.2: expressing (Equation 3) per joint as ${\ddot{q}}_{i}=\delta_{h,i}$ , with $\delta_{h}=M^{-1}\left(q\right)J^{⊤}\left(q\right)f_{\text{ext}}^{b}$ , provides the disturbed double-integrator model on which the bank of ISM observers operates.

#### 2.3.2 Suboptimal second-order sliding mode control

For active trajectory tracking in CTT and HJTT task, a model-free *Suboptimal Second-Order Sliding Mode* (SSOSM) controller is deployed (Bartolini et al., 1998a), identified by Equations 4–6.

For a manipulator with n=7 degrees of freedom, let $q_{d},{\dot{q}}_{d}\in R^{n}$ denote the desired reference trajectory profiles. For each joint i∈{1,…,n} , the sliding variable $\sigma_{i}\in R$ (forming the sliding vector $\sigma ={\left[\sigma_{1},\ldots ,\sigma_{n}\right]}^{T}\in R^{n}$ ) is defined as:

$$\sigma_{i}=\beta_{i}\left(q_{i}-q_{d,i}\right)+\left({\dot{q}}_{i}-{\dot{q}}_{d,i}\right)$$ (4)

where $\beta_{i}=1$ for all joints i∈{1,…,n} (such that $\beta \in R^{n}$ ). The switching gain scaling factor $\alpha_{i}\in R$ (belonging to $\alpha \in R^{n}$ ) is dynamically updated based on the detection of the local extrema $\sigma_{M,i}\in R$ according to the update law formulated in (Bartolini et al., 1998b), with the tuning parameter set to $\alpha_{i}^{*}=0.5$ for all joints. An intermediate discontinuous control input $w_{i}\in R$ is computed as:

$$w_{i}=-\alpha_{i}W_{i}\text{sgn}\left(\sigma_{i}-\frac{1}{2}\sigma_{M,i}\right)$$ (5)

where $W_{i}\in R_{>0}$ is a modulation gain derived from the maximum expected disturbance bound, set to $F_{\text{max},i}=150\text{ N}\cdot \text{m}$ for each joint i (with $F_{\text{max}}\in R^{n}$ ).

Crucially, to eliminate torque chattering, the control torque command $\tau_{\text{cmd},i}\in R$ applied to joint i is generated by continuous time-integration of $w_{i}$ :

$$\tau_{\text{cmd},i}\left(t\right)=\int_{0}^{t}w_{i}\left(\tau \right)\text{ d}\tau \;\;\Rightarrow \;\;{\dot{\tau }}_{\text{cmd},i}\left(t\right)=w_{i}\left(t\right)$$ (6)

This integration step yields a smooth, chattering-free joint torque vector $\tau_{\text{cmd}}={\left[\tau_{\text{cmd},1},\ldots ,\tau_{\text{cmd},n}\right]}^{T}\in R^{n}$ that guarantees finite-time convergence σ→0 independently of the robot dynamic model.

### 2.4 Patient-robot interaction paradigm

Across all operational tasks (CO-PTP, CTT, and HJTT), the AS-ULDAR platform operates in different interaction regimes. During the execution of the CO-PTP task, the system does not provide active force fields, haptic augmentation, or Assistance-As-Needed (AAN) guidance, operating strictly in an unassisted regime. The same paradigm is applied during the recording phase of the CTT task, in which the therapist can manually guide the robot end-effector with no resistance or assistance.

Conversely, during the trajectory reproduction phase for both CTT and HJTT, the robot operates in a fully assisted paradigm. In this regime, no voluntary motor effort is required from the patient, whose limb is fully guided along the prescribed spatial and temporal trajectory by the robot end-effector.

Since the primary scope of this work is conceived as a pilot feasibility study, we deliberately opted to keep the robotic control framework as straightforward and transparent as possible, without implementing complex adaptive force fields. However, integrating adaptive AAN paradigms and patient-cooperative impedance control represents a primary direction for future developments.

### 2.5 Target population, user roles, and clinical rehabilitation workflow

To effectively evaluate the operational feasibility of the integrated platform in a hospital environment, it is essential to define the target clinical population and delineate the operational roles of the two main user groups involved in the system: *Physiotherapists/Occupational Therapists* and *Neurological Patients*.

#### 2.5.1 Target patient population and inclusion criteria

The proposed platform is specifically designed for upper-limb neurorehabilitation and motor-cognitive assessment in adult patients suffering from neurological impairments. The clinical inclusion criteria established for the target population strictly comprise adult individuals aged between 18 and 80 years with a confirmed clinical diagnosis of a chronic neurological condition. Specifically, the system targets patients affected by stroke, Parkinson’s Disease (PD), Amyotrophic Lateral Sclerosis (ALS), or Mild Cognitive Impairment (MCI). Furthermore, patients must present a mild-to-moderate upper-limb motor or functional impairment, quantitatively defined by a QuickDASH (*Quick Disabilities of the Arm, Shoulder and Hand*) score ranging between 20 and 90 points.

#### 2.5.2 User roles and functional division

The platform uses a collaborative approach where tasks are divided based on each user’s role and clinical needs. Physiotherapists and occupational therapists act as the primary operators, serving as the supervisors and configurators of the system. Their operational tasks include manually guiding the robotic arm to record therapeutic trajectories customized to the specific needs of the patient. They are also responsible for initiating the trajectory reproduction under CTT or HJTT tasks and launching the CO-PTP tasks. Additionally, therapists play a crucial role in progress monitoring and parameter tuning, as they interpret the intuitive real-time plots and metric summaries provided by the GUI to adapt difficulty levels, and task parameters based on the patient’s immediate performance.

Conversely, neurological patients are the target end-users who interact directly with the robotic arm to carry out the physical therapy. Their involvement consists of actively performing the CO-PTP task, in both Traditional Mode (TM) and Cognitive Exercise Mode (CEM), during which the robot remains passive and the patient drives the motion in an unassisted paradigm. Once the custom motions have been recorded and mapped by the therapist, patients transition to the fully assisted, robot-guided execution of the CTT and HJTT trajectory-tracking tasks, in which the robot actively guides the patient’s limb along the prescribed trajectory.

#### 2.5.3 Clinical rehabilitation workflow

The clinical protocol foresees an intervention structured over 4 weeks, with a frequency of three sessions per week. Robotic therapy is integrated into a comprehensive care plan, administered alongside conventional physical therapy. The robotic workflow follows a sequential structure: after initial patient setup and profiling on the GUI, the session begins with a baseline motor-cognitive assessment where the patient completes multiple repetitions of the CO-PTP tasks in both Traditional Mode (TM) and Cognitive Exercise Mode (CEM). The therapist then demonstrates and records a custom therapeutic motion. Finally, the session concludes with the guided execution of multiple repetitions of the trajectory tracking exercise, while the therapist maintains high-level supervision to monitor progress and adjust task parameters in real time.

It is worth noting that, while the platform is designed to support this comprehensive workflow, and an ongoing clinical trial is currently collecting data on neurological patients to assess therapeutic efficacy, the present paper focuses exclusively on evaluating system integration, operational feasibility, and therapists-centered usability when the complete platform operates in a clinical setting.

### 2.6 System feasibility and usability evaluation

The aim of this section is to present the methodologies used to evaluate the integrated AS-ULDAR platform from both a technical reliability and usability perspective. While the underlying software architecture, force observer performance, and HJTT algorithm accuracy have been validated in our prior works (Alessi et al., 2025a; 2026; 2025b), this study focuses on the overall system usability, operational feasibility, and technical reliability when deployed in a clinical environment. The objective of this evaluation is not to assess patient-level clinical efficacy, but rather to investigate the quality of user interaction, system stability, and the feasibility of adopting the platform in real-world clinical workflows.

To address the three Research Questions outlined in Section 1, the methodology is structured across three complementary evaluation dimensions: first, technical reliability, operational stability, and execution safety monitoring address **RQ1**; second, a comparative System Usability Scale (SUS) assessment between the standalone GUI and the fully integrated robotic platform addresses **RQ2**; and third, qualitative feedback gathered from end-user physiotherapists captures usability barriers and design guidelines to address **RQ3**.

All procedures involving human participants were conducted in accordance with the ethical standards of the institutional research committee and with the 1964 Helsinki Declaration and its later amendments. Ethical approval for the clinical evaluation was obtained under protocol number [57875/2025]. It is worth noting that all physiotherapists involved in the evaluation had no prior experience with robotic systems. Prior to the experimental session, each participant received approximately 30 min of training covering system operation, robot startup procedures, and the main functionalities of the graphical user interface.

#### 2.6.1 Technical reliability and operational stability assessment

To address **RQ1**, technical reliability and operational feasibility were evaluated concurrently during the usability testing sessions conducted with naïve users in the laboratory and physiotherapists in the hospital setting. Rather than running separate benchmark trials, system stability and software-hardware integration were monitored in real time while participants operated the platform through the complete rehabilitation workflow. Across all usability sessions, technical performance was evaluated across three primary dimensions: system crashes, adverse technical events, and task completion rates.

#### 2.6.2 Usability study

To evaluate user interaction and operational acceptance, a formative usability study was conducted to address **RQ2** and **RQ3**. The objective was not to assess the clinical effectiveness of the rehabilitation exercises, but rather to investigate the quality of user interaction and the practical feasibility of adopting the system in a clinical environment. Since the overall user experience is shaped by both software and hardware components, the evaluation was intentionally structured into two complementary phases. The first phase focused exclusively on the graphical user interface (GUI) to assess software usability independently from the physical robot. The second phase involved physiotherapists operating the complete system in a realistic hospital setting, thereby evaluating the usability of the entire clinical workflow. Together, the System Usability Scale (SUS) scores from both phases address **RQ2** by mapping the usability gap between standalone software and full hardware integration, while the qualitative feedback gathered during the clinical phase addresses **RQ3** by uncovering operational barriers and design insights.

##### 2.6.2.1 GUI usability evaluation

The usability of the GUI was evaluated in a controlled university laboratory environment involving five naïve users without specific expertise in robotics, rehabilitation technologies, or robot programming. Prior to each evaluation session, the robotic platform was fully configured by the researchers. In particular, robot startup, software initialization, and hardware-software communication were completed before participant interaction. Consequently, users were only required to interact with the graphical interface and the functionalities made available through it. Participants were asked to perform a set of representative tasks covering the main functionalities of the GUI:

- creation and selection of patient profiles;
- navigation among the different GUI sections;
- trajectory recording through robotic therapist demonstration;
- configuration of rehabilitation exercises;
- execution of rehabilitation and assessment exercises;
- visualization of exercise results and performance indicators.

At the end of the experimental session, participants completed the System Usability Scale (SUS) questionnaire.

##### 2.6.2.2 Usability evaluation in a clinical environment

The usability of the complete AS-ULDAR platform was evaluated through a formative study involving seven physiotherapists. The experiments were conducted in the rehabilitation gym of the Istituti Clinici Scientifici Maugeri hospital, where the system was deployed in its intended clinical configuration. Unlike the GUI-only evaluation, physiotherapists interacted with the full rehabilitation workflow, including both software and hardware components. In particular, participants were required to operate the system in conditions closely resembling real clinical practice, starting from system initialization and continuing through the execution of rehabilitation exercises. The set of tasks performed during this evaluation included all the tasks defined in the GUI usability study. In addition, prior to the GUI-related tasks, physiotherapists were also required to perform system-level operational procedures, including:

- robot startup and initialization;
- establishment of the connection with the Franka Control Interface (FCI);
- launch of the ROS two communication framework and GUI node;
- verification of system readiness prior to task execution.

At the end of the evaluation, participants completed the System Usability Scale (SUS) questionnaire and provided qualitative feedback through two open-ended questions, as described in the following subsections.

##### 2.6.2.3 System usability scale

The usability of both the GUI and the complete AS-ULDAR platform was assessed using the SUS, a standardized questionnaire widely employed for the evaluation of interactive systems. The SUS consists of ten statements rated on a five-point Likert scale ranging from ‘Strongly Disagree’ to ‘Strongly Agree’ (Brooke, 1996; Grier et al., 2013). Odd-numbered items express positive statements regarding the system, whereas even-numbered items express negative statements. The SUS score was computed according to the standard procedure. For positive items, one point was subtracted from the participant score, while for negative items the participant score was subtracted from five. The resulting values were summed and multiplied by 2.5, yielding a final usability score ranging from 0 to 100. Higher SUS scores indicate better perceived usability. The obtained scores were analyzed separately for the GUI-only evaluation and for the complete-platform evaluation in order to investigate the contribution of software usability and overall system usability to the user experience.

##### 2.6.2.4 Qualitative feedback collection

In addition to the SUS questionnaire, physiotherapists participating in the clinical evaluation were asked to provide qualitative feedback regarding the proposed rehabilitation platform through two open-ended questions. The first question focused on the graphical user interface; the second question concerned the overall rehabilitation platform. Such questions are reported in Table 4. Their objective was to identify potential usability issues, gather suggestions for future developments, and collect clinician perspectives regarding the practical adoption of the platform in rehabilitation settings. The collected answers were subsequently analyzed and grouped according to recurring themes.

**TABLE 4:** Topics and corresponding questions.

Topic	Question
GUI Improvement	What suggestions would you provide to improve the GUI?
Full System Improvement	What suggestions would you provide to improve the overall system?

## 3 Results

This section presents the empirical findings obtained from the multi-method evaluation of the AS-ULDAR platform, directly addressing the three Research Questions outlined in Section 1. First, the technical reliability, system stability, and execution safety logs collected across all testing sessions are reported to answer **RQ1**. Second, the comparative System Usability Scale (SUS) scores obtained from the standalone GUI evaluation and the complete platform clinical trial are presented to address **RQ2**. Finally, the thematic qualitative feedback and operational insights gathered from end-user physiotherapists are detailed to answer **RQ3**.

### 3.1 Technical reliability and operational stability assessment

To address **RQ1**, system event logs, automated ROS two node supervisor reports, and supervisor observation sheets were analyzed across all experimental sessions conducted with both naïve laboratory participants and clinical physiotherapists. Throughout the entire evaluation campaign, the integrated AS-ULDAR platform demonstrated complete technical reliability and operational stability.

No system crashes, software freezes, or unhandled ROS two middleware exceptions were recorded during any of the testing sessions. Hardware-software communication via the Franka Control Interface remained stable without unexpected disconnections or loss of real-time control loops. Furthermore, no adverse technical events, or unplanned emergency stop activations occurred during physical interaction with the robotic manipulator. All participants across both evaluation phases successfully completed 100% of the assigned workflow tasks—ranging from system initialization and trajectory demonstration and motor-cognitive evaluation protocols—without requiring manual software overrides, node restarts, or operator-forced system resets.

### 3.2 Usability test

This section reports the results of the usability evaluation of the proposed rehabilitation platform. The analysis includes the System Usability Scale (SUS) scores collected in both the GUI-only and full-system evaluations, as well as the qualitative feedback obtained from the open-ended questions administered to participants.

#### 3.2.1 System Usability Scale

The SUS scores for the GUI-only evaluation and the full-system evaluation are reported in Figure 8.

**FIGURE 8:**
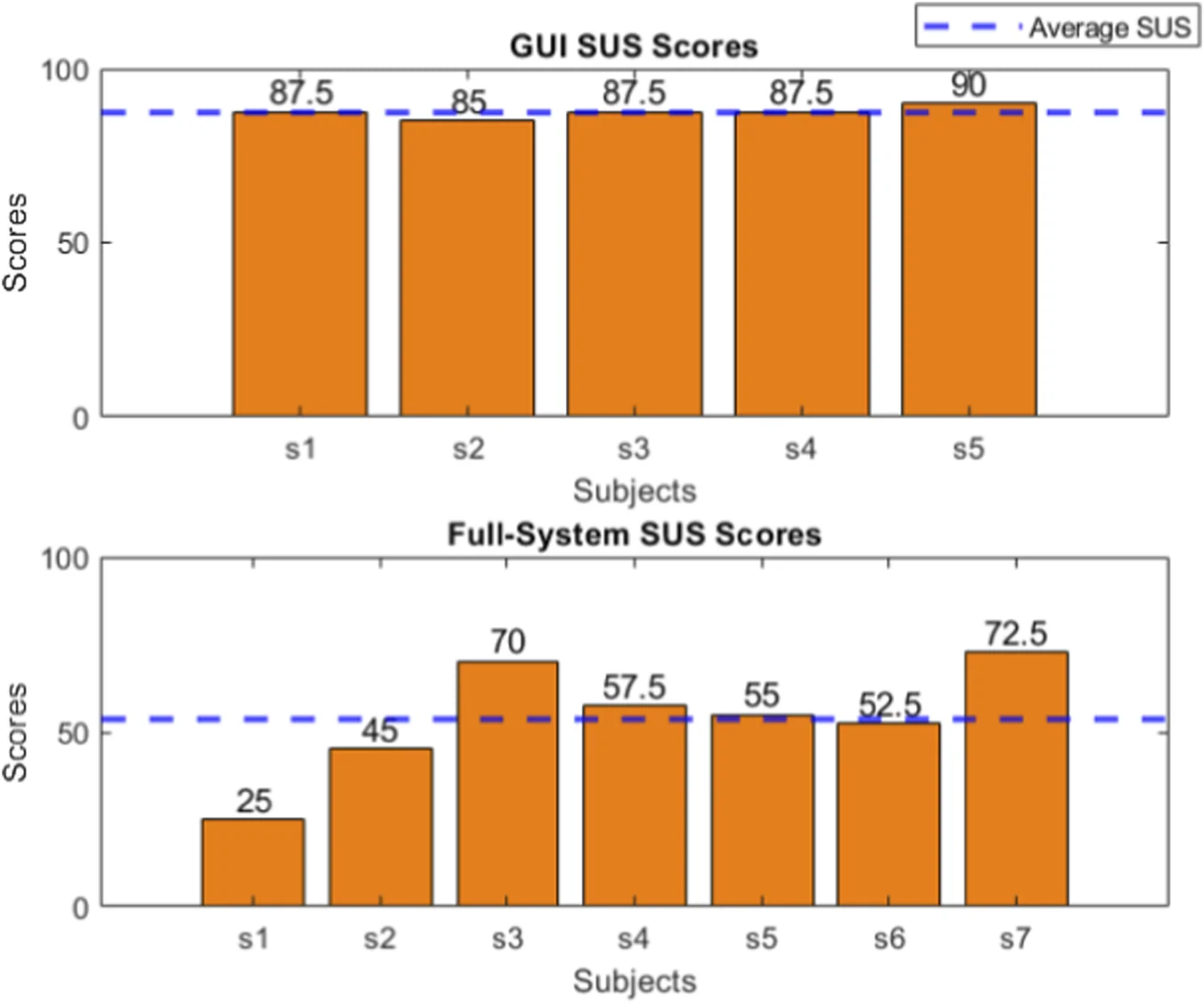
Bar diagram representing the SUS scores regarding the GUI evaluation (top) and the Full-system evaluation (bottom). Each bar represents a different subject, while the horizontal blue line represents the average value.

The GUI-only evaluation yielded high usability scores, with a mean SUS score of 87.5 and a standard deviation of 1.76 across participants.

In contrast, the evaluation of the complete rehabilitation system resulted in a lower mean SUS score of 54, with higher inter-subject variability (standard deviation = 16). The item-level analysis of the SUS responses is reported in Figure 9. For the GUI evaluation, all items achieved mean contributions in the acceptable range (≥2.5) , with the highest scores observed for items 1 (’use frequently’, 3.8 ± 0.4), 3 (’easy to use’, 3.8 ± 0.4) and 5 (’well integrated’, 3.8 ± 0.4). The lowest contribution was recorded for item 9 (’confident’, 2.8 ± 1.2), which also showed the highest inter-subject variability. Overall, the GUI evaluation yielded consistently high contributions across all items, with limited variability among participants. In contrast, the full-system evaluation revealed a more heterogeneous pattern. Items 1 (’use frequently’, 2.86 ± 0.69) and 9 (’confident’, 2.57 ± 0.53) achieved the highest contributions, whereas items 4 (’need support’, 1.14 ± 0.69) and 7 (’learn quickly’, 1.57 ± 0.79) fell in the critical range (<2.0) , indicating that system initialization complexity and the learning curve associated with the robotic platform were the primary sources of usability friction. Items 2 (’complex’, 2.14 ± 1.21), 6 (’inconsistent’, 1.86 ± 0.90), and 8 (’cumbersome’, 2.29 ± 1.11) also showed higher variability, reflecting differences in individual familiarity with robotic systems among the participating physiotherapists.

**FIGURE 9:**
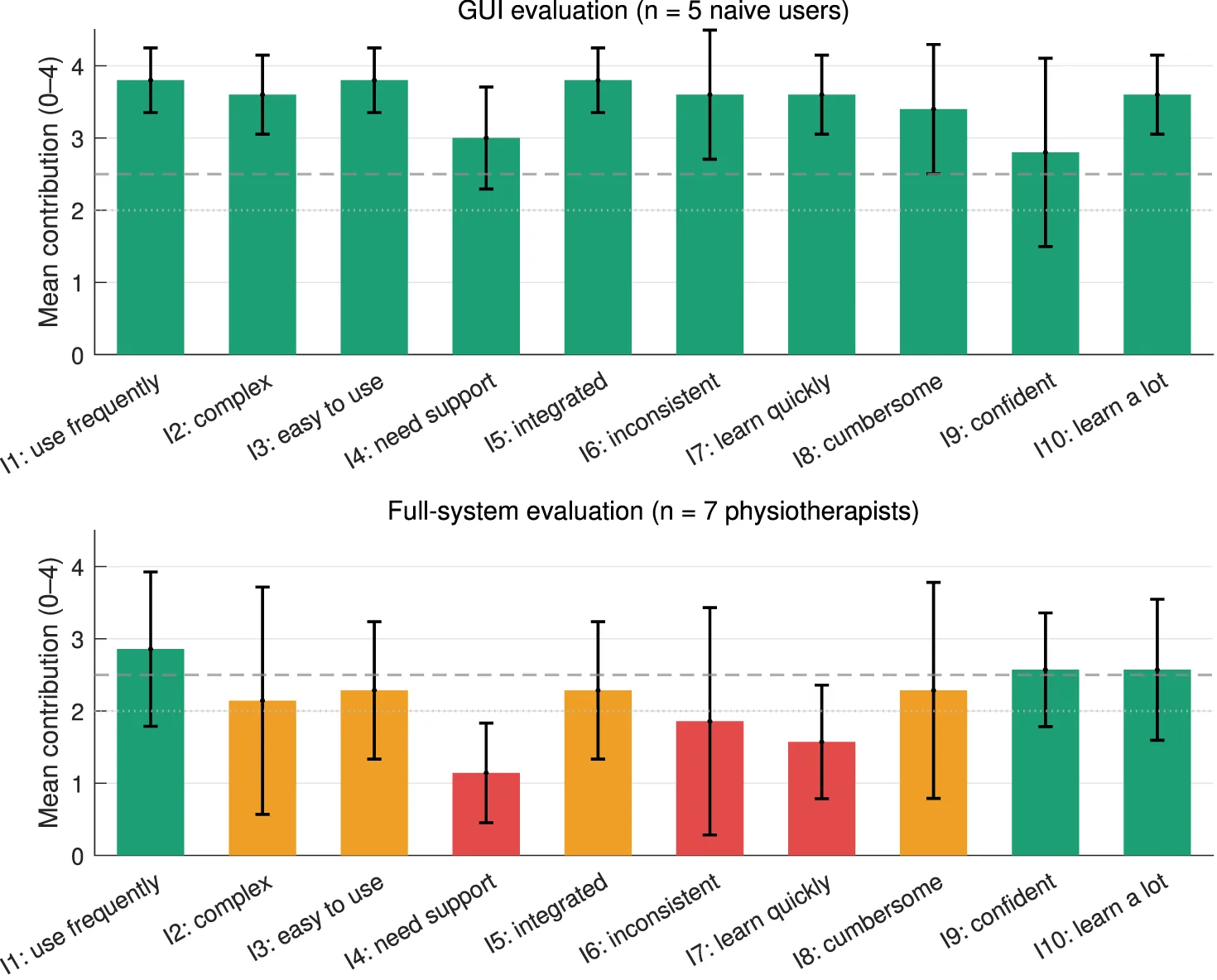
Item-level SUS analysis for the GUI evaluation (top) and the full-system evaluation (bottom). Each bar represents the mean contribution of the corresponding SUS item to the total score (scale 0–4, higher is better), with error bars indicating the standard deviation across participants. Green, amber, and red bars denote items with mean contribution above 2.5 (acceptable), between 2.0 and 2.5 (marginal), and below 2.0 (critical), respectively. The dashed horizontal lines indicate the acceptable (2.5) thresholds.

#### 3.2.2 Qualitative feedback

Qualitative feedback was collected through two open-ended questions addressing improvements for the GUI and for the complete rehabilitation system (Table 4). One of the seven physiotherapists did not provide any suggestion for either question.

##### 3.2.2.1 GUI improvement

Among the remaining six respondents, three explicitly reported no suggestions for improvement, and one indicated insufficient expertise in graphical interface design to provide specific feedback. Overall, five out of seven participants therefore did not raise any substantive concern regarding the GUI. The remaining two responses proposed minor aesthetic or engagement-related enhancements, namely, the introduction of sound-based cues to make the interface more engaging, and a suggestion to make the graphical design more “eco-friendly” in style.

##### 3.2.2.2 Full-system improvement

All six respondents who answered this question proposed at least one operational or physical improvement. Feedback clustered around four recurring themes: (i) intuitiveness of robot motion, particularly for vertical and three-dimensional trajectories, mentioned in three responses, one of which specifically suggested improving the on-screen tutorial for 3D movements; (ii) positioning of the monitor, perceived as slightly lateral rather than fully frontal during task execution; (iii) the range of motion available during rehabilitation exercises, which participants suggested should be expanded; and (iv) the physical footprint of the system, described by one participant as somewhat bulky.

## 4 Discussion

This study presented the technical integration and formative evaluation of the AS-ULDAR platform—an integrated robotic system for upper-limb neurorehabilitation. Rather than evaluating isolated algorithmic modules, this work focused on the systemic contribution of unifying a clinician-oriented interface, sensorless interaction force estimation, motion-capture-based trajectory generation, and motor-cognitive execution tasks within a ROS two architecture. The platform was evaluated to answer three core Research Questions regarding technical reliability (**RQ1**), comparative software vs. full-system usability (**RQ2**), and lessons learned from the usability study (**RQ3**).

### 4.1 Technical reliability and operational stability (**RQ1**)

Addressing **RQ1**, the system demonstrated complete technical stability throughout all experimental trials with laboratory participants and clinical physiotherapists. The 100% task completion rate without software exceptions, middleware freezes, or real-time loop disconnections confirms the viability of ROS 2 Control and Franka Control Interface integration for high-frequency (1 kHz) torque-level interaction. It should be noted that the execution time for each test was relatively brief; therefore, these findings reflect short-term operational viability rather than long-term technical stability or durability.

### 4.2 Comparative usability and confounding factors (**RQ2**)

Regarding **RQ2**, a substantial divergence was observed between the standalone GUI evaluation (mean SUS score 87.5±1.76 ) and the full-system evaluation (mean SUS score 54.0±16.0 ). A plausible interpretation of this outcome is that while software navigation and interface interactions are perceived as highly positive and intuitive, system-level hardware demands—such as physical robot setup, spatial calibration, and real-time exercise execution—introduce significant operational friction and account for the lower, more heterogeneous scores. However, these interpretations must be read with caution, as they are subject to important methodological confounding factors. The two evaluation phases involved distinct participant cohorts, tasks, and experimental settings. The standalone GUI was evaluated by naïve laboratory users performing primarily software navigation tasks, whereas the complete platform was tested by clinical physiotherapists managing physical interaction, setup procedures, and real-world therapy workflows. Consequently, the observed score disparity reflects a combination of participant-related differences (e.g., professional background, prior technical expectations) and task-related operational complexity, rather than a purely causal effect of physical robot demands integration alone.

Regarding the item-level analysis (Figure 9), while the GUI achieved uniformly high scores across all items (all ≥2.5 ), the full-system evaluation revealed severe friction points centered precisely on operational onboarding:

- High Dependency on Support and Steep Learning Curve: The lowest contributions in the full system were recorded for Item 4 (“need the support of a technical person”, mean 1.14/4 ) and Item 7 (“learn to use this system very quickly”, mean 1.57/4 ). These scores clearly reflect the anxiety and technical barrier experienced by clinicians who are experts in therapy but novices with industrial collaborative robots. Brief initial training exposed the vulnerability of expecting clinical staff to autonomously handle middleware startup and hardware initialization without dedicated guided workflows. Consequently, these low scores likely reflect a combination of system-level onboarding complexity and the limited training received by participants;
- Polarized Confidence and Variability: Conversely, items like Item 1 (“use frequently”, mean 2.86/4 ) and Item 9 (“confident”, mean 2.57/4 ) showed that once the system was running, clinicians recognized the therapeutic potential and felt increasingly comfortable. However, the high standard deviation across subjects (SD=16.0) underscores that personal predisposition toward technology heavily dictates whether a clinician perceives the platform as an empowering assistant or an intimidating operational hurdle.

For context, direct comparison with other cobot-based rehabilitation platforms is difficult given the scarcity of SUS scores reported in this domain (Raji et al., 2025). Against general SUS benchmarks, however, a score of 54 falls at the lower end of the “marginal” acceptability range (50–70), below the typical cross-system average of 68 (Bangor et al., 2008), consistent with a first-generation clinical cobot deployment rather than a fundamental usability failure.

### 4.3 Qualitative operational feedback and lessons learned (**RQ3**)

Qualitative feedback collected through open-ended questionnaires further reinforced the operational distinction between GUI usability and full-system physical deployment. GUI-related suggestions were generally minor and non-critical, mostly focusing on optional enhancements such as sound-based targets to augment patient engagement. Conversely, feedback regarding the complete platform highlighted critical physical and interaction-related bottlenecks requiring refinement, including the overall intuitiveness of 3D spatial trajectories, monitor sightline positioning, equipment footprint, and available joint range of motion.

From a design perspective, these findings emphasize that usability in rehabilitation robotics must be treated as a multi-layer property rather than a feature of the software interface alone. Even a well-designed GUI can be severely constrained by system-level factors, such as hardware coordination, startup procedures, physical workspace constraints, and the dual-task demand placed on clinicians who must simultaneously manage therapeutic goals and technical robot operation.

Moreover, several key operational lessons emerge from the qualitative insights. First, physical positioning heavily dictates clinical adoption: the feedback concerning the slightly lateral monitor placement underscores the necessity of co-locating visual feedback directly within the clinician’s primary line of sight toward the patient, avoiding divided attention between monitoring screens and patient safety. Second, extending rehabilitation tasks to complex 3D spatial trajectories requires highly transparent haptic interaction to ensure that physical guidance feels natural and smooth during complex multi-joint movements. Finally, the clinician request for audio-based cues reveals that visual interaction alone is insufficient, highlighting the need for multi-modal (auditory-visual-haptic) feedback to reduce cognitive workload and sustain patient engagement during motor-cognitive training.

Overall, these operational insights demonstrate that the primary barriers to clinical adoption reside within human-robot interaction dynamics, physical spatial integration, and workflow coordination rather than the usability of the graphical interface itself.

### 4.4 Limitations and methodological considerations

Several key limitations must be explicitly considered when interpreting these results:

- Sample Size and Statistical Uncertainty: The sample sizes ( N=5 for GUI, N=7 for full system) align with established usability methodology for exploratory, formative evaluations aimed at identifying the majority of qualitative usability problems (Virzi, 1992; Nielsen et al., 1993). However, small cohorts entail high statistical uncertainty and sensitivity to individual responses (Faulkner, 2003), as reflected by the high standard deviation (SD=16.0) in the full-system SUS score. Therefore, these scores serve strictly as formative feedback to guide iterative system design and cannot support generalized claims regarding definitive system usability or clinical readiness.

Similarly, the HJTT validation cited in support of the platform’s trajectory-tracking functionality (Alessi et al., 2026) was conducted on two healthy subjects, with statistical comparisons based on repeated trials per subject; since such repetitions are not fully independent samples, the corresponding statistical tests may be subject to pseudoreplication, and the reported results should be interpreted as preliminary evidence rather than as a definitive demonstration of superiority over conventional Cartesian trajectory tracking. A broader validation cohort with appropriate mixed-effects statistical modelling is planned as part of future work.

- Absence of Patient Evaluation: A major limitation of this trial is that no neurological patients were included. While the platform is intended for neurorehabilitation, patient usability, acceptability, physical interaction safety, and therapeutic engagement remain unassessed. Translating this system to neurological cohorts will introduce major clinical challenges, including managing patient fatigue, accommodating pathological muscle tone or spasticity, adapting to cognitive/visual impairments, and ensuring active safety during unassisted motor tasks.
- Scope of Usability Assessment: While the SUS provides a global metric of perceived usability, it does not directly capture cognitive workload, physical burden, or operational complexity. Given that the primary bottlenecks identified relate to setup procedures and learning requirements, future studies will incorporate dedicated workload assessment tools (e.g., NASA-TLX) and objective physical interaction metrics to quantify therapist strain.
- Short Session Duration and Long-Term Stability: Each evaluation task was performed over a relatively brief execution window. Consequently, while these preliminary trials demonstrate immediate usability and operational viability, no conclusions can be drawn regarding the long-term technical stability, dynamic performance drift, or operational robustness of the platform over extended clinical use.
- Therapist Training and Familiarity: All participating physiotherapists were naïve to robotic platforms and received only brief onboarding prior to testing. The low scores on SUS Items four and seven directly reflect this initial lack of familiarity. Structured training programs, simplified guided initialization routines, and repeated system exposure are expected to mitigate these setup barriers.

To address these limitations, a formal clinical trial involving neurological patients has received ethical approval (Protocol No. [57875/2025]) and is currently underway. This forthcoming study will evaluate the platform under realistic rehabilitation conditions, combining patient-centered usability, therapist workload metrics, safety protocols for pathological movement, and clinical outcome measures.

### 4.5 Conclusion

Integrating advanced robotic functionalities into a unified platform is technically feasible and operationally stable. However, overall user acceptance is heavily constrained by system setup procedures and workflow complexity rather than software interface design. By identifying these operational bottlenecks in an exploratory setting, this work provides actionable design insights for simplifying robot initialization and spatial ergonomics, paving the way for future clinical effectiveness trials with neurological patients.

## Data Availability

The raw data supporting the conclusions of this article will be made available by the authors, without undue reservation.

## Footnotes

1
https://www.youtube.com/watch?v=leCWvCqveWk
